# Depressive Symptoms Among Older, Sexual Minority Adults and the Influence of Marital Status

**DOI:** 10.1093/geroni/igaa057.539

**Published:** 2020-12-16

**Authors:** Briana Mezuk, Rachel Bergmans, Victoria Schoebel

**Affiliations:** 1 University of Michigan School of Public Health, Ann Arbor, Michigan, United States; 2 University of Michigan Medical School, Ann Arbor, Michigan, United States; 3 University of Michigan, Charlotte, North Carolina, United States

## Abstract

Adverse health outcomes, including poor mental health and depression, tend to be more common among those who are lesbian, gay, or bisexual (LGB). Minority stress theory posits that chronic minoritization contributes to disparities in depression. Factors like social support and income can improve mental health outcomes, and these resilience-promoting factors can be gained through marriage. However, whether marriage improves mental health outcomes in old age regardless of sexual orientation is not well established. This study aims to determine if depressive symptoms differ by sexual orientation in old age, and to test whether the association between depression and sexual orientation was moderated by marital status. The 2016 Health and Retirement Study, a nationally representative sample of U.S. adults older than 50 years (n=4,253), was the first wave to include respondent sexual orientation. Depressive symptoms were measured on the 8-item Center for Epidemiologic Studies Depression Scale (CES-D). Those with a score greater than or equal to 3 were considered to have elevated depressive symptoms. When testing main effects in the adjusted model, depression was not more common among LGB persons than heterosexual persons (OR=0.95; 95% CI=0.46-2.00). Yet, marital status significantly moderated the relationship between sexual orientation and depression (p=0.034). Among heterosexual adults, being married was protective against depression (OR=0.48; 95% CI=0.32-0.71) when compared to being never married, whereas marriage was not protective among LGB adults (OR=0.95; 95% CI=0.26-3.45). Findings indicate that LGB adults do not experience the same mental health benefits of marriage as heterosexual individuals.

